# The Effect of Sleep on Children's Word Retention and Generalization

**DOI:** 10.3389/fpsyg.2016.01192

**Published:** 2016-08-18

**Authors:** Emma L. Axelsson, Sophie E. Williams, Jessica S. Horst

**Affiliations:** ^1^Research School of Psychology, The Australian National UniversityCanberra, ACT, Australia; ^2^School of Psychology, University of SussexBrighton, UK

**Keywords:** word learning, sleep, retention (psychology), generalization (psychology), storybooks, preschool children, infants

## Abstract

In the first few years of life children spend a good proportion of time sleeping as well as acquiring the meanings of hundreds of words. There is now ample evidence of the effects of sleep on memory in adults and the number of studies demonstrating the effects of napping and nocturnal sleep in children is also mounting. In particular, sleep appears to benefit children's memory for recently-encountered novel words. The effect of sleep on children's generalization of novel words across multiple items, however, is less clear. Given that sleep is polyphasic in the early years, made up of multiple episodes, and children's word learning is gradual and strengthened slowly over time, it is highly plausible that sleep is a strong candidate in supporting children's memory for novel words. Importantly, it appears that when children sleep shortly after exposure to novel word-object pairs retention is better than if sleep is delayed, suggesting that napping plays a vital role in long-term word retention for young children. Word learning is a complex, challenging, and important part of development, thus the role that sleep plays in children's retention of novel words is worthy of attention. As such, ensuring children get sufficient good quality sleep and regular opportunities to nap may be critical for language acquisition.

## Introduction

Sleep provides children with numerous benefits (e.g., Touchette et al., 2007; Fondell et al., 2011; Jansen et al., 2011) and is associated with both healthy brain development and neuropsychological functioning (e.g., Dahl, 1999; Siegel, 2005; Hill et al., 2007; Galland et al., 2012). One area of early development that particularly benefits from sleep is language acquisition. Several studies demonstrate that sleeping shortly after exposure to new words or new word forms (e.g., *cathedruke*) contributes to the integration of words into the existing lexicons of adults (e.g., Dumay and Gaskell, 2007, 2012) and school-aged children (e.g., Brown et al., 2012; Henderson et al., 2012), though wakeful rest can also benefit **retention** (e.g., Dewar et al., 2014). Napping shortly after exposure to words also helps toddlers abstract the structure of an artificial language (e.g., Gómez et al., 2006; Hupbach et al., 2009, see also Fischer et al., 2006 with adults).

KEY CONCEPT 1 **Retention**

Retention is the recognition of previously-encountered information after a delay. Word retention often refers to the ability to recognize and correctly indicate a previously-seen object in a context different to the one in which it was originally encountered upon hearing the name of that object.

Sleeping shortly after exposure to new material also helps children retain the meanings of individual words. For example, in a recent study exploring word retention, Williams and Horst (2014) read illustrated stories containing novel words to 3-year-old children, who then either napped or remained awake. Children who napped shortly after hearing the new words retained them significantly better than their wakeful peers when tested approximately 2.5 h after hearing the stories and also 24 h after hearing the stories. The benefit of sleep remained significant 7 days after children had heard the stories. Clearly, then, providing children with the opportunity to sleep following exposures to new words has dramatic consequences for children's **word learning**.

KEY CONCEPT 2 **Word learning**

Word learning may refer to many different types of word knowledge and usage, but often refers to forming an association between a word and referent and the gradual strengthening of that association to enable accurate word or referent recognition and/or word production at a later point in time.

## Word learning

Word learning is a complicated process involving a variety of steps from segmenting a new word from the continuous speech stream, to determining the meaning or referent from others' speech (or written text), to encoding and storing information about a new word such that it can be later recalled and retrieved on demand. Although children appear to complete these steps effortlessly, learning even a single new word is difficult—especially when children are learning multiple words in parallel (see e.g., Axelsson and Horst, 2013) as is often the case in word learning from storybooks. In the literature, “word learning” is used to refer to many different types of word knowledge and usage, including retention, **generalization** and production. For our purposes we are restricting this review to word retention (recognizing the same word or referent later or again) and word generalization (extending a word to a new referent). Both retention and generalization usually occur at a later point in time than the original learning event (though some studies examine generalization in real-time, e.g., Samuelson and Smith, 2005; Samuelson and Horst, 2007) and research into these processes should occur in a new context to ensure robust learning has occurred. Importantly, retention involves the integration of new information (e.g., individual words and referents) into existing memory networks, while generalization typically involves forming a gist of the word's associations and relationships with multiple items (e.g., extension of a word to similar items from the same category) and therefore requires less focus on the individual exemplars (Stickgold and Walker, 2013). Retention is not necessarily a prerequisite for generalization (e.g., Twomey, 2012).

KEY CONCEPT 3 **Generalization**

Generalization involves extracting the statistical regularity between individual items or in the sequential order of items. In relation to word learning, generalization involves extending the association of a word and its referent to similar items from the same category.

## Sleep and word retention

Sleep plays a key role in enhancing, stabilizing, and integrating new information into existing memory networks (Walker, 2009; Stickgold and Walker, 2013), facilitating recall of newly-encoded information at a later point in time (Wilhelm et al., 2013). There are copious studies demonstrating the importance of sleep in the integration of new information and the strengthening or consolidation of new information, henceforth referred to as “**memory consolidation**” (e.g., Diekelmann et al., 2009 for reviews; Stickgold and Walker, 2013), but there are fewer studies explaining the underlying mechanisms of sleep-related learning. A number of studies provide evidence of the particular sleep stages that enhance specific types of memory, such as implicit or procedural skill memory and declarative memories for explicit information accessible to consciousness, for instance, facts, episodes, and semantic information, such as word meanings (e.g., Walker, 2009; Stickgold and Walker, 2013). Adult sleep is characterized by 90 min sleep-stage cycles made up of four non-rapid eye movement NREM sleep stages: Stage 1, Stage 2, Stage 3 slow wave sleep (SWS), Stage 4 (SWS), and rapid eye movement (REM, see Dement and Kleitman, 1957; Hill et al., 2007). These sleep stages are apparent in infants from 6-months of age, but the sleep cycles typically last 50–60 min (Jenni et al., 2004) gradually increasing to 75 min at 2-years (Louis et al., 1997) and 90 min at 6-years (Montgomery-Downs et al., 2006). In adults, REM sleep aids in retention of weak semantic associations (Stickgold et al., 1999) and greater sleep spindle activity in Stage 2 sleep is associated with enhanced integration of novel word forms of previously acquired words, such as *cathedruke*, into existing lexicons (Tamminen et al., 2010). In children, greater Stage 2 sleep spindle activity is associated with memory for object locations (Kurdziel et al., 2013). More precisely, Kurdziel et al. (2013) found nap-related benefits in memory for object locations particularly amongst habitual nappers when naps occurred within 3–5 h of exposure. The naps were largely characterized by SWS, and the increase in performance was positively related to Stage 2 sleep spindle density. There was no evidence of sleep-related benefits in the children who did not nap—even following overnight sleep. Interestingly, proportions of SWS, a stage that is important for declarative memory (see below), remain largely the same across the first 2-years of life, while REM decreases and Stage 2 increases (Louis et al., 1997). Therefore, changes in the durations of sleep stages in the early years could have implications for different developmental domains.

KEY CONCEPT 4 **Memory consolidation**

Memory consolidation refers to the strengthening and enhancement of information encoded. Sleep-related memory consolidation is specific to consolidation of information encountered prior to sleep, such that recognition, retention or generalization is superior to that seen after an equivalent period of time awake.

The neural mechanisms of sleep-related learning in children is unclear, but hypotheses surrounding sleep-related learning in adults are well-documented. Consistent with the finding that sleep is an active rather than a passive state (Dement and Kleitman, 1957), one explanation for the consolidation of new material during sleep is the Active System Consolidation (ASC) theory (see Diekelmann and Born, 2010; Feld and Diekelmann, 2015, for reviews). According to ASC theory, the cortical and hippocampal neurons that are active during encoding are reactivated during sleep. Declarative memories, such as the memory of novel words, are largely reactivated during SWS (Stages 3 and 4). The hippocampus is a temporary repository and plays a driving role in the repetitive replay of new information during SWS, which leads to the gradual strengthening of connections in the relevant cortical areas for long-term storage. The temporary hippocampal representations are eventually “downscaled” and removed. As the sleep stages are cycled through repeatedly, each time SWS is re-entered, the memory traces are reactivated. Therefore, overnight sleep can have better memory strengthening effects than shorter sleep durations (Diekelmann and Born, 2010). SWS typically occurs during the earlier portions of sleep compared to later portions and given the relationship between SWS and declarative memory (Walker and Stickgold, 2006), this could explain the beneficial effects of napping on children's retention of novel words.

Following children's initial encoding of novel words, the associations between words and referents are fragile (McMurray et al., 2012; Munro et al., 2012). Thus, when it comes to child word learning it is likely that there are weak representations in the hippocampus and cortical areas relevant for object, auditory, lexical, and multimodal associative representations (e.g., Rodríguez-Fornells et al., 2009; Robinson and Sloutsky, 2010). During SWS sleep these areas are likely reactivated and following repeated reactivations, the synaptic connections in the cortex are strengthened for long-term storage. Importantly, however, the associative representations are still malleable during tests of retention and the associative representations can be reconsolidated or altered during repeated exposures in subsequent periods of wakefulness (Walker, 2009).

**Figure 1:**
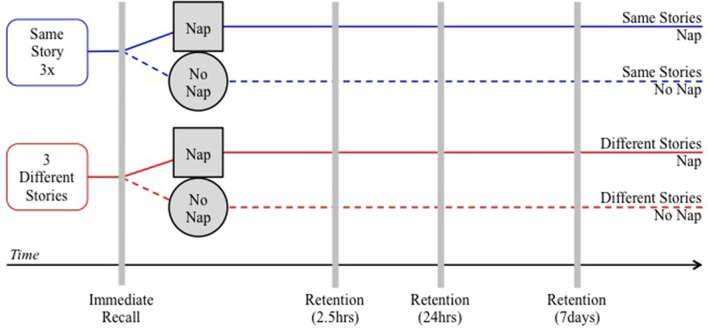
**Schematic of the design in Williams and Horst (2014)**.

Recently, Williams and Horst (2014) demonstrated that 3-year-old children remember more words introduced through storybooks if the children nap soon after storybook reading (within 30–45 min) than if they do not nap. An experimenter visited children in their preschools to either read the same story three times consecutively or three different stories and then tested the effect of sleep on children's ability to retain new vocabulary from those stories (see Figure 1 for a schematic of the study design). The illustrated storybooks depicted novel objects that were incidentally named throughout the story. In both conditions children received the same number of exposures to the target words during the reading phase. First, the experimenter tested immediate recall for the novel names by asking children to point to the correct target object among four decontextualized pictures of novel objects. Next, children who habitually napped took their nap as usual and children who did not habitually nap remained awake but did not hear any more stories. Then, the experimenter tested retention after children awoke. Nap and Wake children were yoked so that the time from the reading phase to the first retention test was the same for both partners. Finally, the experimenter returned 24 h later as well as 1 week later to repeat the retention tests. Note, preschool children generally sleep for equivalent amounts of time within 24 h periods, so even when children no longer nap, they tend to have longer nocturnal sleep (Ward et al., 2008; Lam et al., 2011; Kurdziel et al., 2013).

**Figure 2:**
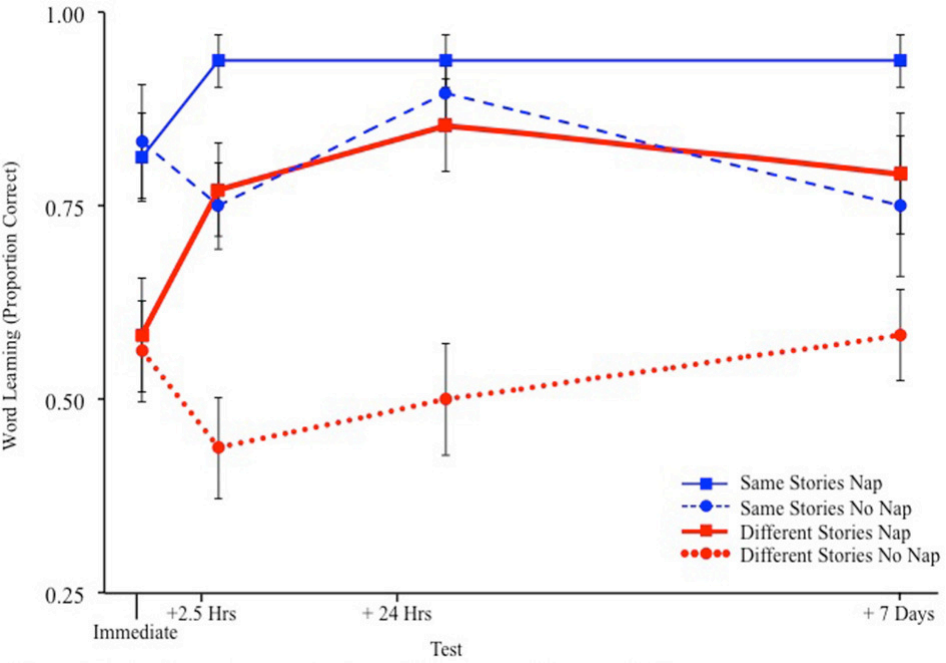
**Word learning results from Williams and Horst (2014)**.

Napping shortly after hearing new words aided retention for 3-year-old children up to 1 week later (see Figure 2). Note, the immediate word learning accuracy scores did not differ for children who later went on to nap or stay awake in either story condition. That is, there were no differences present between the sleep and wake groups immediately after exposure to the stories. Further, the inclusion of this baseline measure helped to tease apart whether sleep is improving children's memory for word meanings or whether or not an equivalent period awake is leading to forgetting (see, Stickgold, 2013 for a similar argument). As can be seen in the figure (comparing baseline to 2.5 h later): both memory consolidation and forgetting during wakefulness are occurring. In particular, reading before nap time was especially helpful for children who were presented with the new words in multiple, different stories—a learning situation already shown to be very difficult compared to hearing the same story repeatedly (Horst et al., 2011). Napping soon after reading allowed children who faced this difficult word learning situation (synthesizing word meanings from across different stories) to perform as well as their peers who heard the same stories but remained awake.

Independent of the effect of sleep, hearing the same (repeated) stories as opposed to hearing different (non-repeated) stories accounted for 41% of the variance in retention scores 1 week later. This advantage for contextual repetition replicates previous research in shared storybook reading (Robbins and Ehri, 1994; Sénéchal, 1997; Horst et al., 2011; McLeod and McDade, 2011; Wilkinson and Houston-Price, 2013) and associative word learning (e.g., Axelsson and Horst, 2014) and is the subject of a separate focused review (i.e., Horst, 2013).

Recently, Horváth et al. (2015) also found a benefit for retention following sleep. Specifically, 16-month-old toddlers who napped within 90 min of exposure to two novel name-object pairs looked longer at the correct targets at test than peers who received the same training but stayed awake. In general, daytime naps are positively associated with greater vocabulary growth for this age group (Horváth and Plunkett, 2016).

Sleeping shortly after exposure to new information enhances memory (Talamini et al., 2008; Payne et al., 2012). However, it is sleep itself rather than time of day (Gais et al., 2006) or time between exposure and sleep (e.g., Seehagen et al., 2015) that affects memory consolidation. Backhaus et al. (2008) trained 9- to 12-year-old children on lists of noun pairs in both the morning and the evening (i.e., before sleep). Children were significantly more accurate at cued recall for words they had learned in the evening than for words they had learned in the morning (see also, Ashworth et al., 2014). However, recall improved following sleep for both morning and evening training. Similarly, Henderson et al. (2012) trained 9-year-old children on lists of pseudo-words either in the morning or the evening. Children who learned the words in the evening (i.e., before sleep) were significantly more accurate on a recognition task than children who had learned the words in the morning. As in the Backhaus et al. (2008) study, performance improved for both groups following sleep and both groups continued to do well 1 week later. In another test of pseudo-word learning, (Brown et al., 2012) found 7-year-old children's performance on cued recall tests also improved following sleep. In this case, children's cued recall was significantly better after a longer delay that included overnight sleep than after a shorter delay (3–4 h) that did not include sleep. Therefore, sleeping soon after exposure is critical, however this is yet to be investigated in detail and is worthy of future research (but see below and Hupbach et al., 2009).

The evidence for the benefits of sleep for memory consolidation in typically-developing (TD) children is increasing. In children with developmental disorders however, sleep difficulties are prevalent, and whether these difficulties can further impact existing cognitive, language, or behavioral difficulties remains a concern (Wiggs, 2001; Annaz et al., 2011; Kozlowski et al., 2012). Axelsson et al. (2013) found a relationship between sleep duration and vocabulary size in toddlers with Williams syndrome (WS), a population with relatively good language profiles. In contrast, a cross-syndrome study comparing TD children and children with Downs syndrome (DS) and WS, demonstrated that sleep quality was related to attention in TD children, but not those with DS and WS (Ashworth et al., 2015). Therefore, despite the prevalence of sleep difficulties in developmental disorders, this study indicates that sleep quality can impact on TD children's potential to attend to and encode novel words. More information on the impact of sleep difficulties in children with developmental disorders is needed.

Sleep deprivation can have a detrimental effect on the ability to encode and retain new information. Sleep deprivation affects the parts of the brain involved in executive functions such as the prefrontal cortex, anterior cingulate, and posterior parietal systems as well as the hippocampus (see Goel et al., 2009, for a review). Walker and Stickgold (2006) deprived adult learners of sleep for 36 h. Despite having had two nights of sleep before encoding, sleep-deprived participants had poorer retention of a word list compared to controls. Even minimal sleep restriction in school-aged children can affect their neurobehavioral functioning as found by Sadeh et al. (2003). Children whose sleep was restricted for less than an hour for 3 consecutive nights, demonstrated poorer performance in tasks measuring memory, attention, and vigilance than children whose sleep was not restricted. Sleep is clearly important post-learning, but also affects children's subsequent ability to encode new information and strengthen memory representations of previously-exposed information. Given the gradual and protracted nature of word learning over time (McMurray et al., 2012; Bion et al., 2013), regular naps and sufficient nocturnal sleep are likely to have important consequences for long-term retention of novel words. As such, it should be a concern that naps at preschools are increasingly being replaced by more time on curriculum instruction (Kurdziel et al., 2013; Mednick, 2013).

The integration of novel words into long-term memory is clearly important for language acquisition and sleep appears to affect children's retention of novel words. However, another skill that is important for language development is the ability to generalize across instances (i.e., across different members of a category), so that children can flexibly extend their learning to other items and in other contexts (Waxman and Booth, 2000; Twomey et al., 2014). There is evidence to suggest that sleep can also affect participants' capacity to generalize their knowledge of multiple items (Darsaud et al., 2011).

## Sleep and generalization

Generalization occurs when learners extract a gist, detect statistical regularities between individual items or determine a rule in the relationship in a sequence of items (Stickgold and Walker, 2013). Sleep aids in the integration of single items into existing memory networks, but it can also contribute to learners' generalization of the relationship between multiple items (Stickgold, 2013). For example, in tests of memory for word lists, adult participants often experience “false memories” for words absent from the original list if presented with a series of semantically-related words (e.g., falsely remembering “fruit” after seeing “banana, ripe, berry, juice, basket, citrus,” etc., Roediger and McDermott, 1995). Sleep enhances false memories for such semantically-related words (Payne et al., 2009; Diekelmann and Born, 2010). This suggests that sleep reorganizes memory in such a way that a “gist” of the original list is retained (Feld and Diekelmann, 2015). The memory for gist words following sleep is related to the number of specific words participants recognize from the original list, rather than the words that are rated as simply familiar (Darsaud et al., 2011).

Sleep enhances both declarative and non-declarative memory (procedural and implicit), which is less accessible to consciousness, and can aid in the recognition of statistical relationships between items and in rule abstraction (Stickgold and Walker, 2013; Feld and Diekelmann, 2015). For example, following sleep, adults demonstrate better implicit learning of a rule that governs the sequence of a series of different tones (Durrant et al., 2011). There are few studies on the underlying mechanisms of sleep-related learning of generalizations, but the ASC theory is also applicable in the integration of multiple items into memory networks (Stickgold and Walker, 2013). SWS is associated with the implicit learning of tone sequences (Durrant et al., 2011), but REM sleep also promotes the recognition of associations between words in adults (Cai et al., 2009).

In relation to children, it appears that children are better than adults at implicit learning, such as learning a hidden sequence of button presses, particularly after a period of sleep (Wilhelm et al., 2013). Toddlers also show evidence of linguistic rule abstraction following sleep. For example, Gómez et al. (2006) found that infants were able to detect the association between the first and last syllables of a sequence of words in an artificial language even when the middle syllables varied (e.g., PEL-wadim-RUD, PEL-chila-RUD), but more so for the infants who napped within 4 h of exposure to the words. Infants who napped approximately 4 h after exposure to the words failed to demonstrate recognition of the word sequences (Hupbach et al., 2009). Similar to the findings of declarative memory for individual items (e.g., Williams and Horst, 2014; Horváth et al., 2015), the length of time between exposure and napping could influence the degree to which sleep enhances implicit learning of the relationship between words.

The need for napping in the first few years of life coincides with a period of exposure to a vast number of novel words and sleep could be particularly helpful for children's memory consolidation of words as well as the abstraction of the rules involved in the relationship between words and the generalization of the meaning of words to multiple items (Stickgold and Walker, 2013). Recently, Friedrich et al. (2015) found evidence of this with 9- to 16-month-old children. Children were presented with specific word-object pairs and words with several items belonging to particular categories (e.g., eight similar items associated with the word *bofel*). Before napping, children were sensitive to only the specific word-object pairs, but after napping (within 1.5 h of exposure) they also demonstrated retention of words and items from these categories indicating that sleep helps in the generalization of category items (see also Horváth and Plunkett, 2016). Children's category learning was also correlated with Stage 2 sleep spindles.

However, Werchan and Gómez (2014) recently observed a benefit for *remaining awake* in a word generalization study. Thirty-month-old toddlers were exposed to multiple exemplars from three different categories of novel objects and the category names. Different colored backgrounds were also used for each exemplar presentation, which increased the overall variability present in the learning phase. Toddlers were tested on their ability to generalize the new object names to novel, never-before-seen exemplars from the object categories either immediately or 4 h after the initial exposure to the novel objects. Among the toddlers who were tested after 4 h, half of the toddlers napped and half remained awake. Only toddlers who had remained awake successfully generalized the novel names. That is, napping did not facilitate novel name generalization in this task. The authors explain their findings in terms of forgetting facilitating abstraction (see also Vlach et al., 2012). This may be especially critical for young children who are prone to encoding both relevant (e.g., object shape) and irrelevant details (e.g., background color) during a task (Gómez and Edgin, 2015). Thus, remaining awake may be more helpful for generalization than sleep among young children because wakefulness can help one forget irrelevant details (Vlach et al., 2012; Werchan and Gómez, 2014).

How might we explain the discrepancy in findings between studies demonstrating benefits for sleep among 16-month-old children (e.g., Friedrich et al., 2015; Horváth and Plunkett, 2016) and benefits for wakefulness with 30-month-old children (e.g., Werchan and Gómez, 2014) for word generalization? One possibility is that these studies are capturing a period of instability in development. Both Dynamic Systems Theory (Thelen and Smith, 1994) and Overlapping Waves Theory (Chen and Siegler, 2000) argue that children progress through both periods of stability and instability—with periods of instability often occurring before qualitative changes in children's cognitive development. Periods of instability can also be observed within the same children (e.g., Martin et al., 2005; Adolph et al., 2008; Corbetta and Snapp-Childs, 2009). Toddlerhood and the preschool years are periods of rapid change, both in children's physical and cognitive development. It is also a period of change in sleep behavior with children dropping from two to only one nap by about 24-months of age and often giving up daytime sleep altogether by 42-months of age (see Weissbluth, 1995, for a review). Recently, several sleep researchers have argued that foregoing naps is an indication of cognitive maturity (Lam et al., 2011; Kurdziel et al., 2013). That is, children give up their naps when they can handle the cognitive load of up to 12 consecutive hours of various input without specific periods of sleep or rest. In addition, daytime sleep may emit unique benefits for habitual nappers that it does not produce for other children. Like all aspects of development, there is seldom a sudden switch from one stage (e.g., habitual napper) to another (e.g., non-habitual napper). Thus, it makes intuitive sense that children are progressing through a period of cognitive instability precisely as their sleep behavior is changing. This instability may explain some of the discrepancies in the data with children. However, additional research is needed to help us better understand how sleep and wakefulness aid word generalization.

## Improvement over time

Children's word learning performance in the Williams and Horst (2014) study did improve over the course of the week for both children who napped shortly after exposure to the storybooks and those who did not—though napping shortly after hearing the new words was a significant advantage. This pattern of results is consistent with other research showing a testing effect—even in the absence of feedback (e.g., Karpicke and Roediger, 2008). Specifically, repeated testing provides additional opportunities for encoding and retrieval of the information being learned as well as the potential for interference, alteration, or reconsolidation of the memories (Stickgold and Walker, 2007). On this view, each retention test presented to children in the Williams and Horst (2014) study afforded children with an additional opportunity for the strengthening of association between the word-object pairs as well as potential interference. Indeed, children are not neural networks: children's learning does not “turn off” and we should expect some additional changes in associative strength in word-object pairs to also occur on test trials. In fact, the 4-alternative test trials presented by Williams and Horst (2014) were highly similar to *training* trials used in other studies, particularly those using cross-situational learning paradigms (e.g., Suanda et al., 2014). Thus, we should expect memory for the word-object associations to improve over time as a byproduct of repeated testing.

## Conclusions

The effect of sleep on adults' declarative and implicit memory is well-established (Feld and Diekelmann, 2015). The evidence for the effect of sleep on declarative and implicit memory in infants and children is also mounting (Ashworth et al., 2014; Berkowitz et al., 2015; Gómez and Edgin, 2015) and in particular the influence of sleep on word learning. During infancy and early childhood, sleep is typically polyphasic, made up of naps, and nocturnal sleep (Galland et al., 2012) and this is also a period when language acquisition undergoes huge developmental changes (Fernald et al., 2006). Both napping and overnight sleep appear to strengthen children's memory for novel words. Word learning typically progresses slowly changing gradually from initial weak word-object associations that become increasingly stronger over time (McMurray et al., 2012; Bion et al., 2013). This could explain why sleep is of benefit to word learning as sleep consolidation benefits are likely to be especially helpful for fragile memories—such as newly-formed representations of words only recently encountered. Regular exposure to novel words coupled with regular sleep phases are likely to contribute to the integration of novel words into children's lexicons (see e.g., Diekelmann et al., 2009).

More evidence is still needed to understand the underlying mechanisms involved in sleep-related word learning in early childhood, but the evidence so far implicating napping and good quality sleep in word learning needs to be brought into the spotlight for parents, health professionals, and educational policy makers (see also, Staton et al., 2016). Ensuring that children are provided with a structured nap and sleep schedule appropriate to their age is likely to have marked implications on their retention of newly encountered words, but also on their capacity to engage in new learning situations.

## Author contributions

All authors listed, have made substantial, direct and intellectual contribution to the work, and approved it for publication.

### Conflict of interest statement

The authors declare that the research was conducted in the absence of any commercial or financial relationships that could be construed as a potential conflict of interest.
